# Identification of circular RNA BTBD7_hsa_circ_0000563 as a novel biomarker for coronary artery disease and the functional discovery of BTBD7_hsa_circ_0000563 based on peripheral blood mononuclear cells: a case control study

**DOI:** 10.1186/s12014-022-09374-w

**Published:** 2022-11-03

**Authors:** Hanxiao Zhou, Xiongkang Gan, Shu He, Yanjun Wang, Sheng Zhang, Jiaxin Chen, Yaqing Zhou, Can Hou, Lei Hua, Qian Zhang, Enzhi Jia

**Affiliations:** Department of Cardiovascular Medicine, The First Affiliated Hospital of Nanjing Medical University, Guangzhou Road 300, 210029 Nanjing, Jiangsu Province China

**Keywords:** Coronary artery disease, PBMC, BTBD7_hsa_circ_0000563, ChIRP-MS

## Abstract

**Background:**

BTBD7_hsa_circ_0000563 is a novel circRNA and contains conserved binding sites with RNA-binding proteins. However, BTBD7_hsa_circ_0000563 has not been fully studied in coronary artery disease (CAD). We aimed to clarify the diagnostic value and the possible functional role of BTBD7_hsa_circ_0000563 in CAD.

**Methods:**

A total of 276 human peripheral blood mononuclear cell (PBMC) samples were employed. The circularization of BTBD7_hsa_circ_0000563 was verified via Sanger sequencing. The expression level of BTBD7_hsa_circ_0000563 in CAD samples and control individuals was analysed via qRT–PCR. The diagnostic potential of BTBD7_hsa_circ_0000563 was evaluated using Spearman’s analysis, univariate and multivariable logistic regression analysis, and receiver-operator characteristic (ROC) curve analysis. ChIRP-MS was performed to directly explore the proteins bound to BTBD7_hsa_circ_0000563. Bioinformatic analysis was conducted to investigate the possible functions and interactions of proteins bound to BTBD7_hsa_circ_0000563.

**Results:**

In the present study, BTBD7_hsa_circ_0000563 was verified as a circular RNA in the PBMCs of CAD patients. The expression level of BTBD7_hsa_circ_0000563 in the CAD group was significantly lower than that in the control group. The area under the ROC curve was 0.690. ChIRP-MS found seven proteins that were directly bound to BTBD7_hsa_circ_0000563. Bioinformatic analysis of these seven proteins showed that the mitophagy and DNA repair pathways were enriched. These proteins interacted with each other to a certain extent.

**Conclusion:**

BTBD7_hsa_circ_0000563 may be a novel biomarker for the diagnosis of CAD and may influence the initiation and progression of CAD. These studies may reveal new possibilities for the diagnosis and treatment of CAD.

**Supplementary Information:**

The online version contains supplementary material available at 10.1186/s12014-022-09374-w.

## Background

Coronary artery disease (CAD) is still the most prominent cause of mortality globally, and it leads to a substantial medical and economic burden worldwide [1]. As a complex chronic disease, CAD is considered to be caused by an interplay between genetic and lifestyle factors [2]. Although the lives of most CAD patients can be saved by the extensive use of drug therapy, percutaneous coronary intervention (PCI), and coronary artery bypass graft (CABG) surgery, the long-term prognosis of CAD and the quality of life for CAD patients are not reported to be improved because early diagnosis is difficult [3]. Therefore, a stable, convenient, sensitive, and specific biomarker is urgently needed, and the deeper mechanisms of CAD need to be explored.

Circular RNAs (circRNAs) are a class of endogenous RNAs with a covalently closed structure. They are generated by backsplicing and are characterized by stability and abundance [4]. Due to the stability and conservation of circRNAs, they have been identified as novel biomarkers for human cancer, diabetes, and Alzheimer’s disease [5–7]. However, the relationship between circRNAs and CAD has been a largely underexplored domain. Interestingly, in our previous study, we found that the BTBD7_hsa_circ_0000563 expression level was negatively associated with the severity of atherosclerosis in human coronary artery specimens [8].

Peripheral blood mononuclear cells (PBMCs) are mononuclear cells in blood and are a key part of the body’s immune system [9]. Inflammatory activation is a key procedure in the development of CAD [10]. Recently, the expression level and diagnostic value of circRNAs for CAD have been explored via PBMC samples [11].

Chromatin isolation by RNA purification (ChIRP) is a technique that can map genome-wide RNA occupancy, and the technique depends on affinity capture of the target RNA-chromatin complex by means of tiling antisense oligos. Therefore, the complex can generate a map of genomic binding sites that has high sensitivity, low background, and a resolution of approximately several hundred bases [12]. Recently, ChIRP has been applied to circular RNA (circRNA) for the understanding of RNA–protein interactions on a genomic scale [13].

In this investigation, we sought to explore the expression patterns and the RNA–protein interaction profile of BTBD7_hsa_circ_0000563 in PBMCs of CAD patients and control individuals. Statistical analysis and bioinformatic analysis were conducted to evaluate the diagnostic value of BTBD7_hsa_circ_0000563 and determine possible associations between CAD and this circRNA.

## Methods

### Study subjects

From October 2020 to June 2021, 238 CAD patients and non-CAD control individuals were recruited for Sanger sequencing and qRT–PCR to verify the circularization and expression of BTBD7_hsa_circ_0000563. Then, from March 2021 to April 2021, 18 CAD patients and 20 non-CAD control individuals were recruited for ChIRP-MS to identify protein–RNA interactions on a genomic scale. From May 2022 to June 2022, 3 CAD patients and 3 non-CAD control individuals were recruited for western blot. All subjects were enrolled at the First Affiliated Hospital of Nanjing Medical University. The enrolled subjects were all older than 18 years of age and had not undergone PCI or CABG before this hospitalization. All subjects underwent coronary artery angiography during this hospitalization. They were categorized into the CAD group (stenosis of the major epicardial coronary artery ≥ 50%) and the control group (stenosis of the major epicardial coronary artery < 50%) according to the ACC/AHA classification [14]. The general exclusion criteria included subjects with congenital heart disease, rheumatic valvular disease, myocardiopathy, cerebrovascular disease, malignant neoplasms, acute or chronic infectious diseases, adrenal dysfunction, severe liver dysfunction, severe kidney dysfunction and thyroid dysfunction. Detailed clinical, demographic, haematologic, and angiographic examinations were performed for all the subjects enrolled in the present study. The severity of coronary artery atherosclerosis was evaluated by means of the Gensini score, as described previously [15].

All experimental protocols used were conducted in accordance with the Declaration of Helsinki and approved by the First Affiliated Hospital of Nanjing Medical University. Written informed consent was obtained from all subjects or their families.

### PBMC isolation for circularization validation and qRT–PCR assay

Nine millilitres of blood was drawn from subjects who underwent overnight fasting via venipuncture upon admission, and this blood sample was used for circularization validation and qRT–PCR assay. PBMCs were isolated from the middle white monolayer using density gradient centrifugation with Lymphocyte Separation Medium (TBD, Tianjin, China). Then, after a second density gradient centrifugation step, the PBMCs were resuspended and preserved in TRIzol reagent (Invitrogen, Carlsbad, CA, USA) at -80 °C until use.

### PBMC isolation for the ChIRP assay

Nine millilitres of overnight-fasted blood samples or ten millilitres of artery blood samples were drawn from subjects by venipuncture upon admission or artery puncture before coronary angiography, respectively, and these samples used for ChIRP. PBMCs were isolated from the middle white monolayer using density gradient centrifugation with Lymphocyte Separation Medium (TBD, Tianjin, China). Then, after a second density gradient centrifugation step, the PBMC samples were preserved at -80 °C until use.

### RNA isolation and qRT–PCR assay

The protocols for RNA isolation and the qRT–PCR assay were specifically described in our previous publication [8]. Briefly, total RNA from PBMCs was extracted using 200 µl chloroform. It was then precipitated with an equal volume of isopropanol and washed with 1 ml of 75% ethanol. Then, after drying for 5 min, it was dissolved in RNase-free water. The quantity and quality of RNA were evaluated by means of a NanoDrop 2000 (NanoDrop Products, Wilmington, DE).

Using the primers listed in Table 1, a total of 500 ng of RNA was used as a template to prepare cDNA for PCR analysis. SYBR Green Real-time PCR Master Mix (Vazyme, Nanjing, China) in a StepOnePlus (Applied Biosystems) instrument was used to quantify the expression level of BTBD7_hsa_circ_0000563. Glyceraldehyde 3-phosphate dehydrogenase (GAPDH) was used for internal normalization. The relative fold-change was calculated using the 2^−ΔΔCt^ method normalized to GAPDH. All experiments were performed in triplicate. Identification of BTBD7_hsa_circ_0000563 as a circRNA was conducted via Sanger sequencing following PCR.

**Table 1:** The oligonucleotide sequences of the primers for qRT–PCR

	Forward primer	Reverse primer
GAPDH	GTCTCCTCTGACTTCAACAGCG	ACCACCCTGTTGCTGTAGCCAA
BTBD7_hsa_circ_0000563	ATGCTTGCACAAGAAATGGA	GAACATGAATGAGGATAATTAG

GAPDH, glyceraldehyde-3-phosphate dehydrogenase

### Chromatin isolation by RNA purification (ChIRP) assays

The ChIRP assay [16] was conducted to explore the interactions between BTBD7_hsa_circ_0000563 and proteins on a genomic scale. First, four biotinylated tiling DNA oligonucleotide probes targeting the splice-junction sequence of BTBD7_hsa_circ_0000563 were designed and synthesized by Ribobio Co., Ltd. (Ribobio, China) (Probe_1 CATTTCTTGTGCAAGCATGT-/3bio/; Probe_2 TCATCCATTTCTTGTGCAAG-/3bio/; Probe_3 CCATTTCTTGTGCAAGCATG-/3bio/; Probe_4 ATCCATTTCTTGTGCAAGCA-/3bio/). A “split-probe” strategy was conducted to resolve the issue that the precipitation of nonspecific DNA fragments by the oligonucleotide probes may be a potential source of noise in CHIRP [16]. To ensure the specificity and accuracy of the BTBD7_hsa_circ_0000563 probes, *LacZ* probes were used as a negative control, and GAPDH was used as an internal control. qPCR was performed to measure the recovery of target RNAs in each group above.

Eighteen CAD samples and 20 non-CAD control individuals were mixed respectively into one CAD sample and one control sample for an independent experiment. After crosslinking with 1% formaldehyde at room temperature for 10 min, PBMCs were lysed using lysis buffer. Subsequently, chromatin from the lysed PBMCs was collected and then hybridized with different tiling probe pools of BTBD7_hsa_circ_0000563 at 37 °C for 4 h with shaking. Afterwards, complexes were incubated with beads conjugated with streptavidin at 37 °C for 30 min. Finally, the chromatin bound to the beads was eluted for LC–MS/MS assays to determine the profile of the proteins bound to BTBD7_hsa_circ_0000563.

### LC–MS/MS assay

The lyophilized peptide fractions were resuspended in ddH2O with 0.1% formic acid. Two-microlitre aliquots of the dissolved sample above were loaded into a nanoViper C18 (Acclaim PepMap 100, 75 μm×2 cm) trap column, after which the trapping and desalting procedure was conducted using 20 µL 100% solvent A (0.1% formic acid). The Easy nLC 1200 system (ThermoFisher) was used to carry out the online chromatography separation. Afterwards, an elution gradient of 5–38% solvent B (80% acetonitrile, 0.1% formic acid) over 60 min was used on an analytical column (Acclaim PepMap RSLC, 75 μm×25 cm C18-2 μm 100 Å). The tandem MS data were acquired on a ThermoFisher Q Exactive mass spectrometer (ThermoFisher, USA) fitted with a Nano Flex ion source, of which the ion spray voltage was 1.9 kV and the interface heater temperature was 275 °C. Data-dependent acquisition (DDA) mass spectrometry techniques were used for MS scanning and tandem MS data acquisition. For the MS1 scan, the scan ranged from 350 to 2,000 m/z at a resolution of 70,000 and a maximum injection time of 100 ms. For the MS2 scan, only spectra with a charge state of 2–5 were selected for fragmentation by higher-energy collision dissociation with a normalized collision energy of 28. The MS2 spectra were acquired in the ion trap in rapid mode with an AGC target of 8,000 and a maximum injection time of 50 ms. Dynamic exclusion was set for 25 s.

The MS/MS data were analysed for the identification and quantification of proteins bound to BTBD7_hsa_circ_0000563 using PEAKS Studio 10.6. The false discovery rate (FDR) was set < 0.7% after searching against the target database with a maximum of two missed cleavages. The settings of oxidation (M), acetylation (protein N-term), deamidation (NQ), pyro-glu from E, pyro-glu from Q for variable modifications and fixed carbamidomethylation of cysteine were selected. The precursor and fragment mass tolerances were set to 10 ppm and 0.05 Da, respectively.

The mass spectrometry proteomics data have been deposited to the ProteomeXchange Consortium via the PRIDE [17] partner repository with the dataset identifier PXD033196.

### Western blot

Protein samples for western bolt were extracted from PBMC cell lysates. RIPA buffer (Beyotime, China) was used for protein extraction according to the manufacturer’s instructions. The protein concentration was determined by the BCA method. The protein lysate was separated by SDS-PAGE and transferred to a PVDF membrane. The membrane was soaked in 5% milk and incubated with the primary antibody at 4℃ overnight. The primary antibodies against UBB (1:1000) and the control protein, β-actin (1:7500) were from proteintech and affinity, China. Horseradish peroxidase-labeled IgG (1:15000) was used as the secondary antibody (abcam, USA). Enhanced chemiluminescence kits (Thermofifisher, USA) were used for signal development. ImageJ software was used to analyze the grayscale values.

### Statistical analysis

Categorical data are presented as counts (percentages). Continuous variables conforming to a normal distribution are described as the mean ± standard deviation, and skewed distribution variables are described as the median (25th–75th interquartile range). Student’s *t test* and the Mann–Whitney U test were used to compare the demographic and clinical pathological characteristics and the circRNA expression level between CAD patients and control individuals. The chi-square test was used to compare the categorical data between two groups. Spearman’s correlation analysis was conducted to explore conventional cardiovascular risk factors and environmental factors related to BTBD7_hsa_circ_0000563. Univariate and multivariable logistic regression analyses were performed to determine whether BTBD7_hsa_circ_0000563 could be an independent factor for CAD. The receiver-operator characteristic (ROC) curve was used to evaluate the value of circRNA as a diagnostic biomarker for CAD. All data were analysed using SPSS 21 software. Two-tailed *P* values < 0.05 were considered statistically significant.

### Bioinformatics analysis

Gene Ontology (GO) functional enrichment and Kyoto Encyclopedia of Genes and Genomes (KEGG) pathway analyses of the proteins bound to BTBD7_hsa_circ_0000563 were performed using the clusterProfiler 4.0 package in R [18]. Protein–protein interaction (PPI) analysis was performed using the STRING database (STRING v11.0) (https://string-db.org/). Interaction prediction scores between proteins and BTBD7_hsa_circ_0000563 were obtained from RNAct database (https://rnact.crg.eu/).

## Results

### Identification of BTBD7_hsa_circ_0000563 as a circRNA

**Fig. 1:**
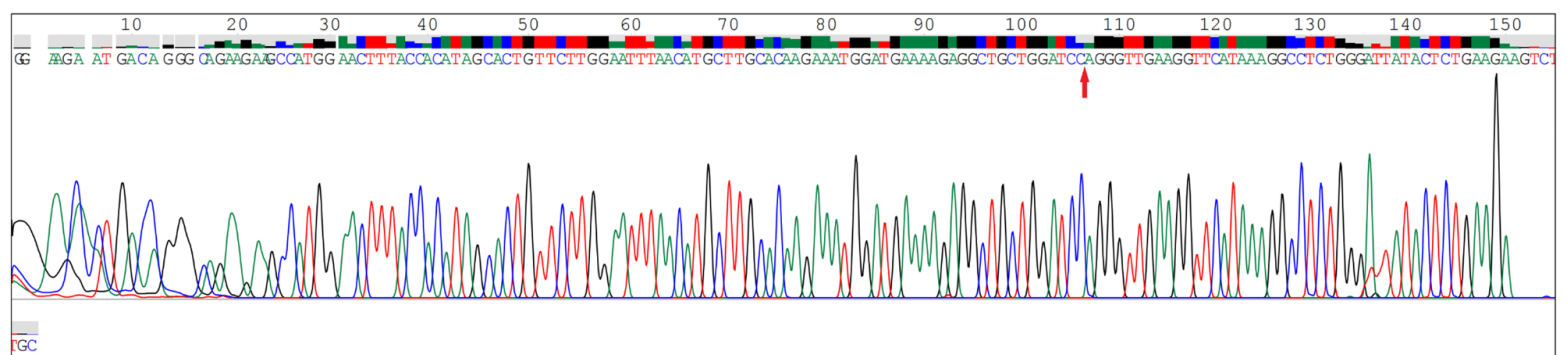
The cyclization site confirmed by Sanger sequencing

To verify that BTBD7_hsa_circ_0000563 is a circular RNA, we designed divergent primers that specifically amplified the back-spliced forms of BTBD7. The “head-to-tail” splicing of BTBD7_hsa_circ_0000563 in the PBMCs of 4 CAD patients was confirmed via Sanger sequencing (Fig. 1). Moreover, the clinical and demographical characteristics of subjects undergoing BTBD7_hsa_circ_0000563 circularization validation are presented in Table 2.

**Table 2:** Baseline characteristics of the subjects grouped according to various populations

Characteristic	Circularization set	Expression set			CHIRP-MS set		
	**CAD (n = 4)**	**CAD (n = 210)**	**Control (n = 24)**	***P*** **value**	**CAD (n = 18)**	**Control (n = 20)**	***P*** **value**
Age (years)	65.25 ± 3.95	65.00 (57.00–72.00)	65.00 (61.00–69.00)	0.970	70.00 (53.50-76.25)	63.00 (55.25–69.25)	0.443
Sex (male/female)	3/1	155/55	11/13	0.004	12/6	9/11	0.210^#^
BMI (kg/m^2^)	23.21 ± 4.76	24.72 (22.86–27.04)	25.34 (22.86–26.67)	0.582	25.01 ± 3.61	25.35 ± 3.04	0.777
SBP (mmHg)	139.25 ± 24.19	130.66 ± 16.92	129.50 ± 16.55	0.751	130.00 ± 17.60	131.53 ± 13.05	0.768
DBP (mmHg)	82.75 ± 8.34	77.00 (70.00–85.00)	78.00 (73.00–88.00)	0.212	75.53 ± 11.75	79.79 ± 10.04	0.249
Hypertension (n, %)	1 (25.0)	134 (63.81)	15 (62.50)	0.899	15 (83.3)	7 (35.0)	0.007^#^
Diabetes (n, %)	0 (0)	53 (25.24)	5 (20.83)	0.636	0 (0)	0 (0)	——
Smoking (n, %)	2 (50.0)	85 (40.48)	9 (37.50)	0.778	10 (55.6)	5 (25.0)	0.099^#^
Drinking (n, %)	2 (50.0)	63 (30.00)	4 (20.83)	0.349	11 (61.1)	5 (25.0)	0.049^#^
TC (mmol/L)	4.16 ± 0.34	3.54 (2.91–4.44)	4.01 (3.41–4.63)	0.233	3.60 ± 0.67	4.15 ± 0.92	0.043
TG (mmol/L)	1.56 ± 0.81	1.42 (0.98–1.84)	1.16 (0.85–1.62)	0.250	1.27 (1.08–1.52)	1.11 (0.88–1.22)	0.098
HDL-C (mmol/L)	1.24 ± 0.26	0.97 (0.86–1.15)	1.12 (0.97–1.26)	0.060	1.04 ± 0.28	1.22 ± 0.32	0.082
LDL-C (mmol/L)	2.42 ± 0.41	2.02 (1.56–2.66)	2.21 (1.93–2.75)	0.319	1.98 (1.58–2.63)	2.29 (1.96–2.88)	0.065
Fasting blood glucose (mmol/L)	4.96 ± 0.46	5.21 (4.56–6.20)	5.17 (4.59–5.95)	0.963	4.95 (4.89–5.21)	5.05 (4.73–5.72)	0.704
Serum creatinine (umol/L)	87.53 ± 26.07	71.90 (63.68–85.10)	67.60 (53.00-78.20)	0.062	69.65 (62.50-79.88)	61.00 (50.23–72.70)	0.051
Gensini score	52.13 ± 29.85	40.00 (22.00–77.00)	1.25 (0.00-4.75)	< 0.001	30.50 (10.00-64.75)	0.00 (0.00-2.38)	< 0.001

Data are expressed as the mean ± standard deviation, median (25th–75th interquartile range), or count (percentage)

#: Fisher’s exact test

CAD, coronary artery disease; BMI, body mass index; SBP, systolic blood pressure; DBP, diastolic blood pressure; TC, total cholesterol; TG, triacylglycerol; HDL-C, high-density lipoprotein cholesterol; LDL-C, low-density lipoprotein cholesterol

### Validation of BTBD7_hsa_circ_0000563 expression levels

**Fig. 2:**
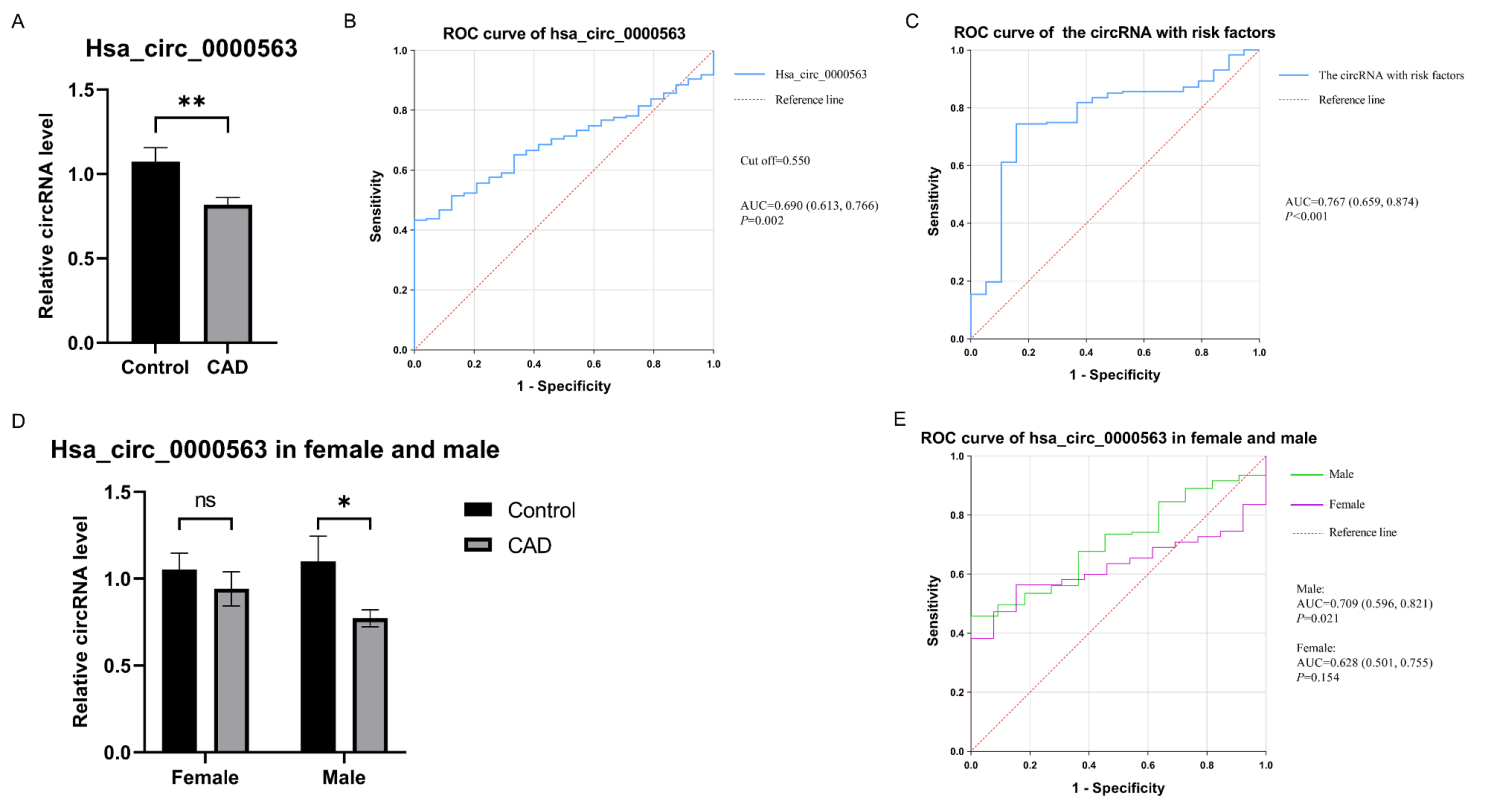
The expression level and the ROC curve of BTBD7_hsa_circ_0000563. (**A**) The expression level of BTBD7_hsa_circ_0000563 in the CAD group was significantly lower than that in the control group (**: *P* < 0.01). (**B**) ROC curve analysis of BTBD7_hsa_circ_0000563. (**C**) ROC curve analysis of BTBD7_hsa_circ_0000563 with conventional risk factors. (**D**) The expression level of BTBD7_hsa_circ_0000563 stratified based on sex (*: *P* < 0.05, ns: *P* > 0.05). (**E**) ROC curve analyses of BTBD7_hsa_circ_0000563 based on sex. CAD, coronary artery disease; ROC, receiver-operator characteristic; AUC, area under the receiver-operator characteristic curve

To verify the connection between BTBD7_hsa_circ_0000563 levels and CAD, the expression level of BTBD7_hsa_circ_0000563 in PBMCs was measured via qRT–PCR in a large population (210 CAD patients and 24 control individuals). The clinical and demographical characteristics of the samples undergoing BTBD7_hsa_circ_0000563 expression validation are presented in Table 2. The expression level of BTBD7_hsa_circ_0000563 in the PBMCs of CAD subjects and control individuals is presented in Table 3. In general, BTBD7_hsa_circ_0000563 showed significantly lower expression levels in CAD patients than in control individuals (*P* = 0.002, Fig. 2 A).

**Table 3:** The expression levels of BTBD7_hsa_circ_0000563 in the validation population

circRNA	CAD (n = 210)	Controls (n = 24)	Fold change	*P* value
BTBD7_hsa_circ_0000563	0.65 (0.34–1.10)	1.02 (0.74–1.33)	0.64	0.002

Data are expressed as the median (25th–75th interquartile range)

CAD, coronary artery disease

Given the difference in sex distribution between the CAD group and control group, we stratified the data based on sex and analysed it again. In males, the expression level of BTBD7_hsa_circ_0000563 in the CAD group was still significantly lower than that in the control group (P = 0.020, Fig. 2D). However, in females, the expression level of the circRNA was not significantly different between the two groups (*P* = 0.158, Fig. 2D). Nevertheless, the expression level of BTBD7_hsa_circ_0000563 still showed a downwards trend in female CAD patients.

### Correlations between BTBD7_hsa_circ_0000563 expression levels and the clinical and demographical characteristics

To test whether the expression level of BTBD7_hsa_circ_0000563 was correlated with cardiac risk factors and conventional CAD diagnostic markers, we conducted Spearman’s correlation analysis. The results (Table 4) showed that the expression level of BTBD7_hsa_circ_0000563 was associated with the Gensini score (*coefficient* = -0.130, *P* = 0.046), which indicates that BTBD7_hsa_circ_0000563 might participate in the progression of CAD.

**Table 4:** Correlations between the baseline characteristics and circRNA levels in the subjects

Parameters	BTBD7_hsa_circ_0000563	
	*Coefficient*	*P* value
Age (years)	0.008	0.901
BMI (kg/m^2^)	-0.076	0.246
SBP (mmHg)	-0.034	0.607
DBP (mmHg)	0.012	0.853
TC (mmol/L)	-0.047	0.477
TG (mmol/L)	-0.106	0.107
HDL-C (mmol/L)	0.039	0.558
LDL-C (mmol/L)	-0.047	0.476
Fasting blood glucose (mmol/L)	0.096	0.143
Serum creatinine (umol/L)	0.010	0.885
Gensini score	-0.130	0.046

BMI, body mass index; SBP, systolic blood pressure; DBP, diastolic blood pressure; TC, total cholesterol; TG, triacylglycerol; HDL-C, high-density lipoprotein cholesterol; LDL-C, low-density lipoprotein cholesterol

### Confirming BTBD7_hsa_circ_0000563 as an independent predictor for CAD

To explore the predictive value of BTBD7_hsa_circ_0000563 for CAD, we divided the subjects into quarters based on the interquartile range of the circRNA expression level. As presented in Table 5, univariate logistic regression analysis showed that the level of BTBD7_hsa_circ_0000563 was inversely associated with CAD without adjustment (*OR* = 0.518, 95% *CI*: 0.334–0.802, *P* = 0.003). After adjusting for the impact of other risk factors, an independent negative correlation between BTBD7_hsa_circ_0000563 and CAD was still observed (*OR* = 0.509, 95% *CI*: 0.304–0.851, *P* = 0.010). These results identify BTBD7_hsa_circ_0000563 as an independent predictor for CAD.

**Table 5:** Univariate and multivariate logistic regression analysis to identify this circRNA as an independent predictor of CAD

Variable	*β*	*SE*	Wald	*OR* (95% *CI*)	*P*
*Univariate logistic regression model*					
BTBD7_hsa_circ_0000563	-0.658	0.223	8.689	0.518 (0.334, 0.802)	0.003
*Multivariate logistic regression model*					
BTBD7_hsa_circ_0000563	-0.676	0.263	6.614	0.509 (0.304, 0.851)	0.010
Sex	-1.732	0.821	4.453	0.177 (0.035,0.884)	0.035

The multivariate logistic regression model included sex, age, BMI, smoking status, drinking status, hypertension, diabetes, TC, TG, HDL, LDL, fasting blood glucose, and serum creatinine

OR: odds ratio; CI: confidence interval

### Diagnostic potential of BTBD7_hsa_circ_0000563

The area under the ROC curve (AUC) for the BTBD7_hsa_circ_0000563 level in predicting CAD was 0.690 (95% *CI*: 0.613–0.766, *P* = 0.002, Fig. 2B). Due to the complex aetiology of CAD, we introduced conventional markers and risk factors for CAD (including sex, age, BMI, smoking status, fasting blood glucose level, and HDL level) into the ROC curve model. After that, the AUC increased to 0.767 (95% *CI*: 0.659–0.874, *P* < 0.001, Fig. 2 C), with a sensitivity of 0.7447 and specificity of 0.8421. These findings may shed light on the value of BTBD7_hsa_circ_0000563 as a biomarker for CAD diagnosis, especially after being combined with conventional markers and risk factors.

Furthermore, we conducted ROC analyses for BTBD7_hsa_circ_0000563 in females and males separately due to the difference in sex distribution. In males, the AUC was 0.709 (95% CI: 0.596–0.821, P = 0.021, Fig. 2E), and in females, the AUC was 0.628 (95% CI: 0.501–0.755, P = 0.154, Fig. 2E). BTBD7_hsa_circ_0000563 showed good diagnostic value for CAD in males, although the diagnostic potential of BTBD7_hsa_circ_0000563 in females remains to be further studied.

### Proteins bound to BTBD7_hsa_circ_0000563

**Fig. 3:**
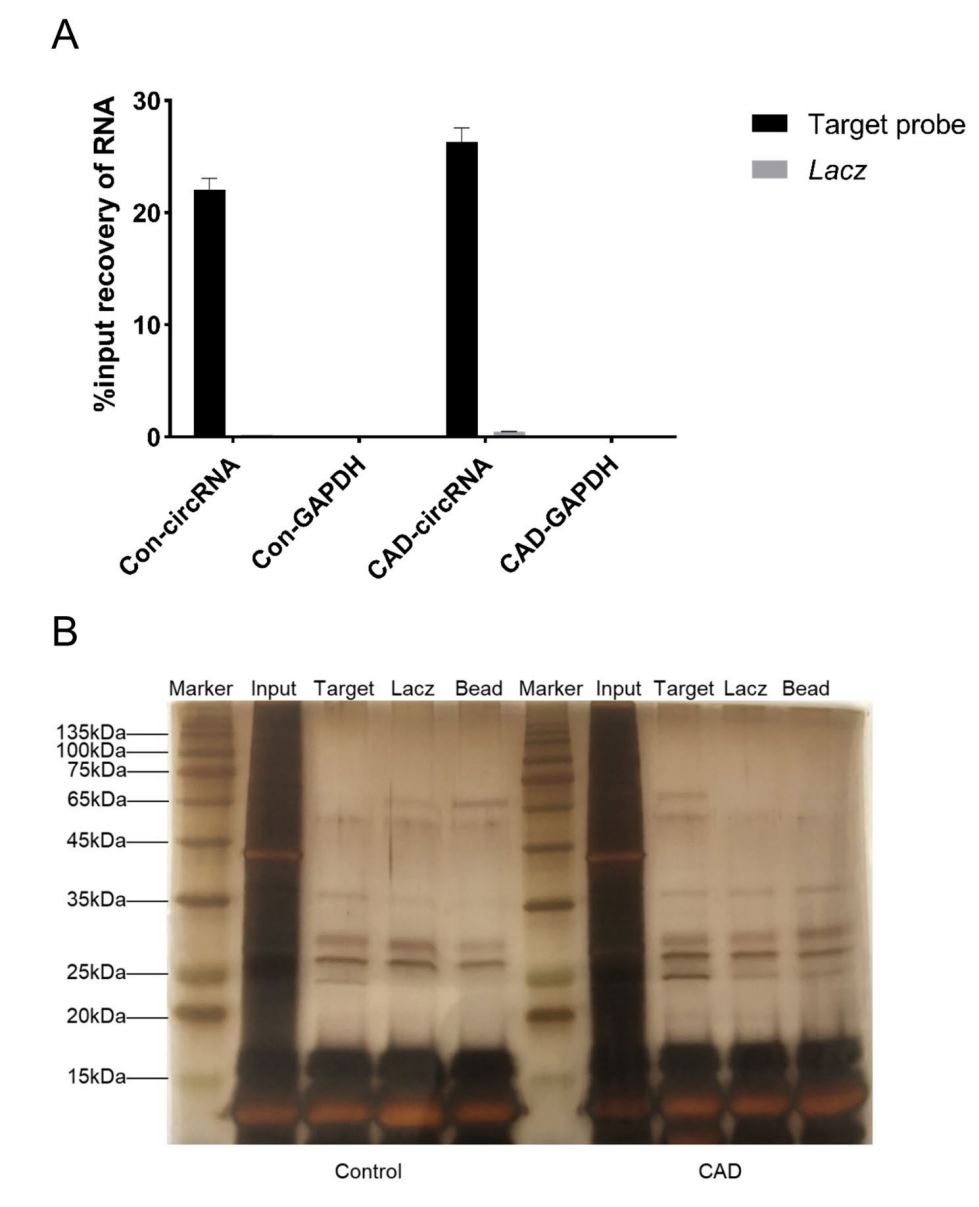
Verification of circRNA probes and ChIRP products (**A**) qRT–PCR results verifying the specificity and accuracy of the BTBD7_hsa_circ_0000563 probes. (**B**) The results of silver staining assay for ChIRP products. CAD, coronary artery disease; ChIRP, chromatin isolation by RNA purification

BTBD7_hsa_circ_0000563 was significantly retrieved from chromatin with the BTBD7_hsa_circ_0000563 probes compared to *LacZ* probes (*P* < 0.001, Fig. 3 A). In addition, the BTBD7_hsa_circ_0000563 probes did not retrieve GAPDH (*P* < 0.001, Fig. 3 A). The results above demonstrated the specificity and accuracy of the probes.

The silver staining assay for ChIRP products, including input, target, Lacz and bead groups, was conducted. The results of silver staining showed that differential bands actually existed between the target groups and all control groups (*LacZ* and bead groups) (Fig. 3B), indicating that the products of the ChIRP assay in the present study are highly specific at the proteome level.

A total of 134 peptides were identified with FDR < 0.7% via PEAKS Studio after the LC–MS/MS assay. The peptide-spectrum matches (PSM) score can evaluate the similarity between actual and theoretical spectra. The distribution of PSM scores (Figure S1a) showed that all error spectra had scores below 33.5. Therefore, we set the filter criteria for peptides to have a PSM score greater than 33.5. Simultaneously, the distribution of mass accuracy (Figure S1b) showed that the mass accuracy of the target spectra was approximately 0, which indicated high accuracy. The analyses above can guarantee the reliability of the peptide.

After removing contaminating proteins, such as keratin and serum albumin, seven proteins were identified to interact with BTBD7_hsa_circ_0000563 directly through the ChIRP-MS assay (Table 6). Six proteins were harvested from the 20 PBMC samples of control individuals, and 1 protein was harvested from the 18 PBMC samples of CAD patients.

**Table 6:** Proteins bound to BTBD7_hsa_circ_0000563

Group	ID	Protein name	-10lgP
CAD	P05165|PCCA_HUMAN	Propionyl-CoA carboxylase alpha chain, mitochondrial	37.66
Control	P0CG47|UBB_HUMAN	Polyubiquitin-B	41.77
	P62987|RL40_HUMAN	Ubiquitin-60 S ribosomal protein L40	41.77
	P62979|RS27A_HUMAN	Ubiquitin-40 S ribosomal protein S27a	41.77
	P0CG48|UBC_HUMAN	Polyubiquitin-C	41.77
	P07339|CATD_HUMAN	Cathepsin D	40.85
	P05089|ARGI1_HUMAN	Arginase-1	37.40

CAD, coronary artery disease

### GO enrichment analysis

**Fig. 4:**
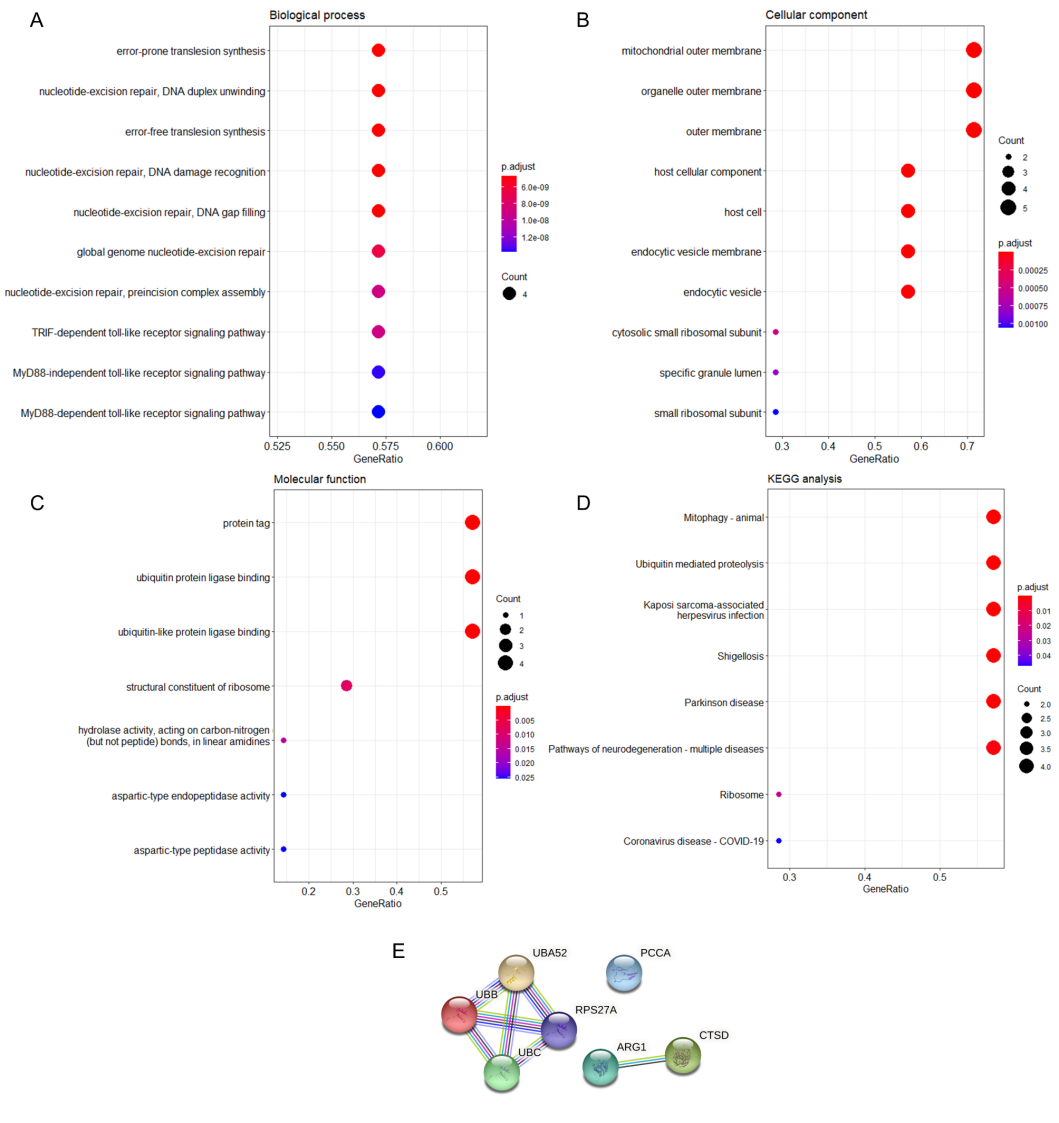
Bioinformatic analyses of proteins bound to BTBD7_hsa_circ_0000563. (**A**) The top 10 biological process terms of Gene Ontology (GO) analysis for proteins bound to BTBD7_hsa_circ_0000563. (**B**) The top 10 cellular component terms of GO analysis for proteins bound to BTBD7_hsa_circ_0000563. (**C**) The 7 molecular function terms of GO analysis for proteins bound to BTBD7_hsa_circ_0000563. (**D**) The Kyoto Encyclopedia of Genes and Genomes (KEGG) analysis results of proteins bound to BTBD7_hsa_circ_0000563. (**E**) The protein–protein interaction (PPI) networks of proteins bound to BTBD7_hsa_circ_0000563. UBB, polyubiquitin-B; UBC, polyubiquitin-C; UBA52, ubiquitin-60 S ribosomal protein L40; RPS27A, ubiquitin-40 S ribosomal protein S27a; ARG1, arginase-1; CTSD, cathepsin D; PCCA, propionyl-CoA carboxylase alpha chain mitochondrial

To further explore the possible biological and functional activities of the 7 proteins bound to BTBD7_hsa_circ_0000563, GO analyses were conducted with adjusted *P* < 0.05. In accordance with the GO enrichment results, 199 biological process terms, 27 cellular component terms, and 7 molecular function terms were enriched. The results demonstrated that the top three biological processes in which these proteins participated included error-prone translesion synthesis, nucleotide-excision repair, DNA duplex unwinding, and error-free translesion synthesis (Fig. 4 A). The cellular component of these proteins was mainly derived from the mitochondrial outer membrane, organelle outer membrane, and outer membrane (Fig. 4B). Furthermore, in the molecular function category, these proteins were involved in protein tag, ubiquitin protein ligase binding, and ubiquitin-like protein ligase binding (Fig. 4 C).

### KEGG enrichment analysis

In accordance with the KEGG enrichment analysis, the proteins bound to BTBD7_hsa_circ_0000563 were involved in a total of 8 pathways. The eight enriched pathways were mitophagy – animal, ubiquitin mediated proteolysis, Kaposi sarcoma-associated herpesvirus infection, Shigellosis, Parkinson disease, pathways of neurodegeneration - multiple diseases, ribosome, and coronavirus disease - COVID-19 (Fig. 4D).

### Protein–protein interaction networks

To further explore the potential interactions between the proteins bound to BTBD7_hsa_circ_0000563, PPI prediction was performed (Fig. 4E). The PPI network showed 7 nodes and 7 edges, in which ubiquitin-60 S ribosomal protein L40, ubiquitin-40 S ribosomal protein S27a, polyubiquitin-B, and polyubiquitin-C interacted with each other directly, and arginase-1 also closely interacted with cathepsin D. However, the propionyl-CoA carboxylase alpha chain had no connection with other proteins. These PPI prediction results suggest that the proteins bound to BTBD7_hsa_circ_0000563 cooperated with each other to a certain extent and that the first four proteins listed above were the most closely related proteins.

### Validation of UBB (polyubiquitin-B) expression levels

To find out the hub protein, -10lgP–values and interaction scores of proteins were taken into consideration (Table S1, Additional file 2). Herein, we found 4 possible key proteins including UBB, UBC, RPS27A, and UBA52. In addition, polyubiquitination is a critical modification in mitophagy[19] and Razeghi’s study has confirmed that UBB may be involved in hypoxia-induced cardiac remodeling[20]. Therefore, we speculated that UBB is most likely to be related to BTBD7_hsa_circ_0000563 and cardiovascular diseases among these 7 proteins.

**Fig. 5:**
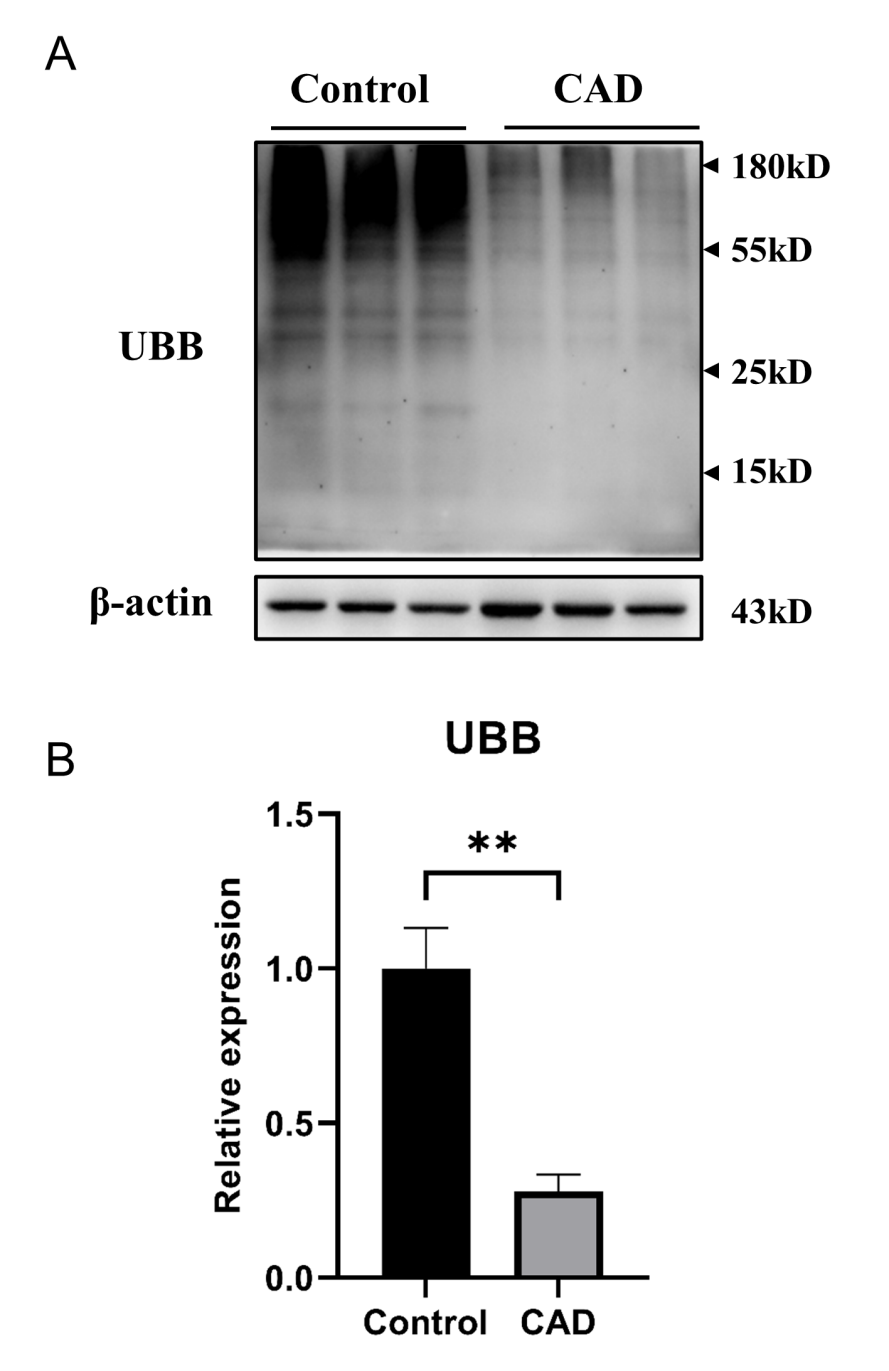
Validation of UBB (polyubiquitin-B) expression levels via western blot (**A**) The representative western blot image. (**B**) The statistical result of grayscale values (**: P < 0.01). The entire lane of each sample was used to determine the grayscale values. CAD, coronary artery disease; UBB, polyubiquitin-B

Western blot was used to investigate the relationship between UBB and BTBD7_hsa_circ_0000563. Obvious accumulation of polyubiquitinated proteins was found in PBMCs of the control group (*P* = 0.007, Fig. 5), implying that UBB (polyubiquitin-B) was positively proportional to BTBD7_hsa_circ_0000563 at a post-transcriptional level.

The clinical characteristics of subjects undergoing western blot are shown in Table S2, Additional file 2.

## Discussion

In this research, we investigated BTBD7_hsa_circ_0000563 in human PBMCs and found that it was relevant to CAD. First, BTBD7_hsa_circ_0000563 was verified as a circular RNA in the PBMCs of CAD patients. We demonstrated that the expression level of BTBD7_hsa_circ_0000563 in PBMCs of the CAD group was significantly lower than that in the control group. Moreover, we identified the diagnostic value of BTBD7_hsa_circ_0000563 with an AUC of 0.690, and its expression level correlated with the Gensini score in CAD patients. A significant negative correlation between the circRNA and CAD was observed via univariate and multivariable regression analysis. In addition, seven proteins were confirmed to bind to BTBD7_hsa_circ_0000563 directly in the PBMCs of CAD patients and control individuals. GO and KEGG enrichment analyses suggested that these proteins that were bound to BTBD7_hsa_circ_0000563 were more concentrated on the biological processes of mitophagy and DNA repair. PPI networks indicated that these proteins cooperated with each other to a certain extent.

Innate immune and inflammatory processes play important roles in the initiation and progression of CAD [21], and PBMCs are key players in these processes [22]. In addition, compared to other human tissues, PBMCs are easier to obtain in actual clinical operations. Therefore, our study was based on human PBMCs, which is rare in research on circRNAs related to CAD. Simultaneously, the present study may also be the first to apply ChIRP-MS, a highly sensitive and specific technique to directly explore RNA binding proteins (RBPs) [16, 23], to the investigation of circRNAs related to CAD.

Memczak et al. first reported BTBD7_hsa_circ_0000563 in animals [24]. BTBD7_hsa_circ_0000563 is located on chromosome 14. The gene code of BTBD7_hsa_circ_0000563 starts at position 93,760,203 and ends at position 93,762,503, and contains conserved binding sites with RNA-binding proteins according to the Circbank database (http://www.circbank.cn/). Thereafter, BTBD7_hsa_circ_0000563 was found in normal human tissues [25–27] and cancer cells [28]. Interestingly, according to our previous study [8], the expression of BTBD7_hsa_circ_0000563 in coronary artery segments with severe atherosclerosis was lower than that in coronary artery segments that were relatively normal. The present investigation further confirmed that BTBD7_hsa_circ_0000563 existed and was circularized in the PBMCs of CAD patients. Having conducted qRT–PCR in a larger population, we verified that the expression of BTBD7_hsa_circ_0000563 in PBMCs of the CAD group was lower than that in PBMCs of the control group. Moreover, we found this circRNA was inversely associated with CAD after adjusting for the impact of conventional risk factors. Specifically, with a 25% increase in the BTBD7_hsa_circ_0000563 level, the risk of CAD occurrence decreased 49.10%. This evidence implies that BTBD7_hsa_circ_0000563 may be a protective factor and an independent predictor for CAD. In addition, in our study, the sensitivity and specificity of BTBD7_hsa_circ_0000563 combined with clinical factors were 0.75 and 0.84, respectively. This finding indicates that the diagnostic value of the combination of BTBD7_hsa_circ_0000563 with clinical factors is better than that of 12-lead electrocardiogram (ECG) (sensitivity: 0.52, specificity: 0.66) [29] and similar to that of coronary computed tomography angiography (CTA) (sensitivity: 0.90, specificity: 0.60) [30]. However, taking the cost and convenience of diagnostic methods into consideration, the levels of BTBD7_hsa_circ_0000563 in PBMCs might serve as a superior new diagnostic strategy for CAD.

For the difference in the diagnostic value of BTBD7_hsa_circ_0000563 between males and females, we speculate that there are two possible reasons. First, the sample size of females was insufficient (significantly less than that of males), and this was a single-centre study. Second, there were significant sex differences in gene regulation [31]. A large-scale multicentre cohort study should be conducted in the future to verify these conjectures.

Furthermore, seven proteins were identified as RBPs of BTBD7_hsa_circ_0000563, including polyubiquitin-B, ubiquitin-60 S ribosomal protein L40, ubiquitin-40 S ribosomal protein S27a, polyubiquitin-C, cathepsin D, propionyl-CoA carboxylase alpha chain mitochondrial, and arginase-1. By means of GO and KEGG enrichment analyses, we found that these proteins were mainly located in the mitochondrial outer membrane and involved in the mitophagy and DNA repair pathways.

The concept of mitophagy was first proposed by Lemasters and was identified as a selective autophagy process that specifically targets damaged or dysfunctional mitochondria [32]. As the byproduct of electron transfer, intracellular reactive oxygen species (ROS) are predominantly produced by the respiratory chain in mitochondria [33, 34]. ROS accumulated over time in cells, ageing and calcium dysregulation can cause mitochondrial damage and dysfunction [35, 36]. Moreover, the study of Jia et al. revealed that mitochondrial dysfunction can affect the occurrence and development of CAD by excessively producing ROS and proton leakage, decreasing the mitochondrial membrane potential, and interfering with the respiratory chain [37]. In contrast, mitophagy can eliminate ageing or dysfunctional mitochondria via receptor-mediated pathways and ubiquitin‐mediated pathways to maintain mitochondrial homeostasis [19]. According to Li et al. and Zheng et al., mitophagy can alleviate the atherosclerotic process by promoting the migration of endothelial cells and reducing the apoptosis of endothelial cells after ox-LDL-induced inflammatory injury [38, 39]. A recent study indicated that mitophagy may inhibit macrophage pyroptosis induced by ox-LDL to reduce atherosclerosis [40]. In addition to mitophagy, DNA repair processes were also markedly enriched. A previous study found that the mean level of DNA damage was significantly higher in CAD patients than in control individuals [41]. Moreover, as a mechanism of DNA repair, the defectiveness of nucleotide-excision repair was confirmed to impel cardiovascular ageing and dysfunction in mice, which may indicate the importance of DNA repair in CAD [42].

The PPI network of the present investigation showed that the proteins bound to BTBD7_hsa_circ_0000563 cooperated with each other to a certain extent, especially the four proteins named ubiquitin-60 S ribosomal protein L40, ubiquitin-40 S ribosomal protein S27a, polyubiquitin-B, and polyubiquitin-C. Ubiquitin-60 S ribosomal protein L40 and ubiquitin-40 S ribosomal protein S27a are both ubiquitin fusion proteins [43]. Polyubiquitin-B and polyubiquitin-C are both ubiquitin polymers [44]. The ubiquitin-mediated pathway is an important mechanism in the induction of mitophagy [19]. Multiple reports have demonstrated that the ubiquitin kinase PINK1 can phosphorylate and activate the E3 ubiquitin ligase Parkin. Parkin can amplify the mitophagy signal generated by PINK1 and ultimately induce mitophagy by polyubiquitination of mitochondrial surface proteins [45–48]. Moreover, ubiquitin phosphorylated at the Thr12 and K63-linked polyubiquitin chains can promote DNA repair [49, 50]. Therefore, these four proteins may be the core proteins. It is worth noting that polyubiquitin-B was positively proportional to BTBD7_hsa_circ_0000563 at a post-transcriptional level. These findings above suggest that the BTBD7_hsa_circ_0000563/polyubiquitin-B axis might be involved in the initiation and development of CAD.

Collectively, all the evidence above suggests that BTBD7_hsa_circ_0000563 may be involved in the initiation and progression of CAD through the mitophagy and DNA repair pathways. These results might provide new ideas for the future study of the potential mechanisms of CAD occurrence and development.

The present investigation had several limitations. First, the sample size was small. The size of these groups was too small to minimize the experimental bias. Thus, for practical application in clinical diagnosis, a large-scale multicentre cohort study is still required to further validate the results obtained from the present study. Second, in addition to RNP, the ChIRP assay can also explore DNA and RNA that bind to BTBD7_hsa_circ_0000563, which is lacking in the present study. ChIRP-DNAseq and ChIRP-RNAseq assays are essential in further investigations to determine the upstream and downstream mechanisms of BTBD7_hsa_circ_0000563 in CAD. Third, only the proteins bound to BTBD7_hsa_circ_0000563 were explored, but the mechanisms in which these proteins take part were merely based on bioinformatic analysis. Therefore, for practical application as therapeutic targets in clinical treatment, in vitro and in vivo experiments of BTBD7_hsa_circ_0000563 and the 7 proteins at the cellular and animal levels should be performed to verify their function and mechanism of action in CAD.

## Conclusion

In conclusion, the present study validated the circularization of BTBD7_hsa_circ_0000563 in the PBMCs of CAD patients and confirmed that the expression level of BTBD7_hsa_circ_0000563 in the PBMCs of the CAD group was significantly lower than that in the control group. The ROC curve and univariate and multivariable logistic regression analyses revealed the value of this circRNA as an independent biomarker for CAD. By means of ChIRP-MS, seven proteins bound to BTBD7_hsa_circ_0000563 were captured. Bioinformatic analysis revealed that these proteins were mainly located on the mitochondrial outer membrane and are involved in the mitophagy and DNA repair pathways. These results indicate that BTBD7_hsa_circ_0000563 and the proteins bound to it may be novel targets for CAD. These studies may provide new ideas for the diagnosis, prevention, and treatment of CAD.

## Electronic supplementary material

Below is the link to the electronic supplementary material.

**Additional file 1: Figure S1** PSM score distribution. (a) Distribution of peptide score. (b) Scatterplot of peptide score versus precursor mass error. Decoy represented error spectrums and Target represented target spectrums. The vertical dotted line represented the threshold of PSM score which was set as filter criteria.

**Additional file 2:** Table S1. Interaction analyses between BTBD7_hsa_circ_0000563 and 7 proteins Table S2. Baseline characteristics of the subjects undergoing western blot.

## Data Availability

The datasets used and/or analysed during the current study are available from the corresponding author on reasonable request.

## List of abbreviations

CAD: coronary artery disease.
PBMC: peripheral blood mononuclear cell.
PCR: polymerase chain reaction.
qRT–PCR: real-time quantitative reverse transcription polymerase chain reaction; ChIRP:chromatin isolation by RNA purification.
ROC: receiver-operator characteristic.
GAPDH: glyceraldehyde 3-phosphate dehydrogenase.
MS: mass spectrum.
FDR: false discovery rate.
AUC: area under the receiver-operator characteristic curve.
PSM: peptide-spectrum matches.
GO: Gene Ontology.
KEGG: Kyoto Encyclopedia of Genes and Genomes.
PPI: protein–protein interaction.
ROS: reactive oxygen species.
PINK1: PTEN-induced putative kinase 1.
