# Supplementary figures and images for: Identification of circular RNA BTBD7_hsa_circ_0000563 as a novel biomarker for coronary artery disease and the functional discovery of BTBD7_hsa_circ_0000563 based on peripheral blood mononuclear cells: a case control study
Source: Clin Proteomics. 2022 Nov 3;19:37. doi: 10.1186/s12014-022-09374-w (PMC9630807; doi:10.1186/s12014-022-09374-w)

A

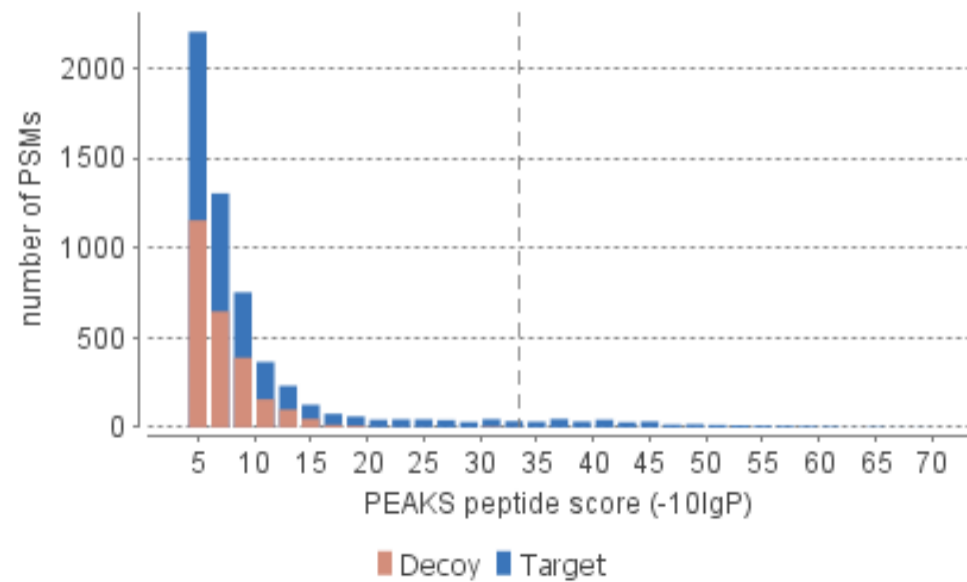

B

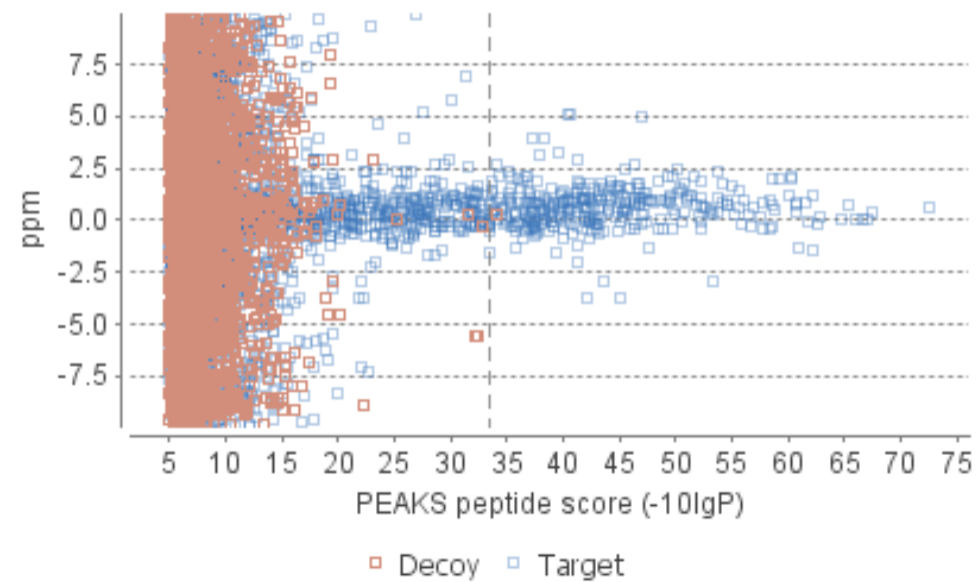

Supplement: Supplementary file 1 — Additional file 1: Figure S1 PSM score distribution. (a) Distribution of peptide score. (b) Scatterplot of peptide score versus precursor mass error. Decoy represented error spectrums and Target represented target spectrums. The vertical dotted line represented the threshold of PSM score which was set as filter criteria. [file 12014_2022_9374_MOESM1_ESM.pdf]
