# Supplementary material for: Identification of circular RNA BTBD7_hsa_circ_0000563 as a novel biomarker for coronary artery disease and the functional discovery of BTBD7_hsa_circ_0000563 based on peripheral blood mononuclear cells: a case control study
Source: Clin Proteomics. 2022 Nov 3;19:37. doi: 10.1186/s12014-022-09374-w (PMC9630807; doi:10.1186/s12014-022-09374-w)
Supplement: Supplementary file 2 — Additional file 2: Table S1. Interaction analyses between BTBD7_hsa_circ_0000563 and 7 proteins Table S2. Baseline characteristics of the subjects undergoing western blot. [file 12014_2022_9374_MOESM2_ESM.docx]

**Table S1. Interaction analyses between BTBD7_hsa_circ_0000563 and 7 proteins**

| Protein name | Gene symbol | LC–MS/MS | STRING | RNAct |
| --- | --- | --- | --- | --- |
|  |  | -10lgP | Node degree | Interaction prediction score |
| Polyubiquitin-B | UBB | 41.77 | 3 | 22.15 |
| Polyubiquitin-C | UBC | 41.77 | 3 | 22.15 |
| Ubiquitin-40S ribosomal protein S27a | RPS27A | 41.77 | 3 | 22.15 |
| Ubiquitin-60S ribosomal protein L40 | UBA52 | 41.77 | 3 | 22.15 |
| Propionyl-CoA carboxylase alpha chain | PCCA | 37.66 | 1 | 27.07 |
| Cathepsin D | CTSD | 40.85 | 0 | 21.23 |
| Arginase-1 | ARG1 | 37.40 | 1 | 21.61 |

**Table S2. Baseline characteristics of the subjects undergoing western blot**

| **Characteristic** | **CAD (n=3)** | **Control (n=3)** | ***P* value** |
| --- | --- | --- | --- |
| Age (years) | 61.67 ± 9.29 | 69.33 ± 16.56 | 0.523 |
| Sex (male/female) | 1/2 | 2/1 | 1.000^#^ |
| SBP (mmHg) | 142.67 ± 15.31 | 135.00 ± 7.81 | 0.483 |
| DBP (mmHg) | 81.67 ± 21.22 | 81.33 ± 11.59 | 0.982 |
| Hypertension (n, %) | 2 (66.67) | 2 (66.67) | 1.000^#^ |
| Diabetes (n, %) | 0 (0) | 1 (33.33) | 1.000^#^ |
| Smoking (n, %) | 0 (0) | 1 (33.33) | 1.000^#^ |
| Drinking (n, %) | 1 (33.33) | 1 (33.33) | 1.000^#^ |
| TC (mmol/L) | 4.64 ± 1.08 | 3.11 ± 0.76 | 0.115 |
| TG (mmol/L) | 2.20 ± 1.31 | 1.56 ± 1.79 | 0.646 |
| HDL-C (mmol/L) | 1.17 ± 0.51 | 1.23 ± 0.52 | 0.893 |
| LDL-C (mmol/L) | 2.90 ± 0.70 | 1.63 ± 0.45 | 0.056 |
| Fasting blood glucose (mmol/L) | 5.29 ± 0.29 | 7.55 ± 4.71 | 0.494 |
| Serum creatinine (umol/L) | 62.97 ± 8.28 | 75.00 ± 20.56 | 0.400 |
| Gensini score | 28.00 ± 14.42 | 3.00 ± 3.61 | 0.044 |

Data are expressed as the mean ± standard deviation or count (percentage).

#: Fisher’s exact test.

CAD, coronary artery disease; BMI, body mass index; SBP, systolic blood pressure; DBP, diastolic blood pressure; TC, total cholesterol; TG, triacylglycerol; HDL-C, high-density lipoprotein cholesterol; LDL-C, low-density lipoprotein cholesterol.
